# Draft Genome Sequence of Byssochlamys sp. Isolate BYSS01, a Filamentous Fungus Adapted to the Fuel Environment

**DOI:** 10.1128/genomeA.00164-18

**Published:** 2018-03-08

**Authors:** Osman Radwan, Thusitha S. Gunasekera, Oscar N. Ruiz

**Affiliations:** a Environmental Microbiology Group, University of Dayton Research Institute, Dayton, Ohio, USA; b Fuels and Energy Branch, Aerospace Systems Directorate, Air Force Research Laboratory, Wright-Patterson AFB, Ohio, USA

## Abstract

Byssochlamys sp. isolate BYSS01 (anamorph, Paecilomyces sp.), which was isolated from jet fuel, is highly adapted to grow in hydrocarbons, having predicted genes involved in degradation of *n*-alkanes, branched alkanes, and aromatic compounds. The draft genome size is estimated at 29 Mb, containing 8,509 proteins.

## GENOME ANNOUNCEMENT

The filamentous fungus Byssochlamys sp. isolate BYSS01 was recovered from Jet A fuel and identified based on morphological characteristics and sequence similarity (94%) to the 18S rRNA gene of Byssochlamys fulva. Byssochlamys sp. (teleomorph) belongs to the genus Paecilomyces, which comprises formaldehyde-resistant and heat-resistant food spoilage fungi (1–4). It was confirmed that the BYSS01 isolate metabolizes alkanes and aromatic hydrocarbons efficiently; this is the first report of a Byssochlamys species adapted to kerosene fuel. Therefore, to gain a deeper understanding of the adaptive mechanisms in isolate BYSS01, its genome was sequenced.

A whole-genome shotgun (WGS) approach on a HiSeq 2000 platform was used to generate TruSeq paired-end libraries, resulting in 47,245,046 paired-end reads (150× sequence coverage), with a read length of 100 bp (∼4.72 Gb). The raw sequences were trimmed using Trimmomatic (5), and reads shorter than 50 bp were removed. The sequence reads were *de novo* assembled with SPAdes software (6), generating a draft assembly comprised of 394 scaffolds greater than 500 bp, with an *N*_50_ value of 463,366, an *L*_50_ value of 20 scaffolds, and a G+C content of 49.3%. BUSCO (7) identified 1,425 out of 1,438 ultraconserved eukaryotic genes in Byssochlamys sp. isolate BYSS01 (99.1%). Repetitive sequences (3.09%) were masked using the RepeatMasker program (8), and the masked genome was used for gene prediction by Augustus 2.5.5 (9) with an option set for Aspergillus oryzae parameters, resulting in 8,509 protein-encoding genes. The average gene density is one gene per 1.67 kb, with an average of 3.45 exons per gene. Approximately 21,244 introns, ranging from 34 to 6,725 bp in length, are present in the genome, with 140 bp as an average intron size and 2.5 as the average number of introns per open reading frame.

A BLASTP search against UniProt and the annotated genomes of Hormoconis resinae, Aspergillus fumigatus, and Byssochlamys spectabilis (4) revealed significant matches (E value, 1 × 10^−5^) for 6,646, 7,578, 7,808, and 7,508 proteins, respectively. Using the Carbohydrate-Active Enzymes (CAZy) database (10), a total of 334 fungal enzymes involved in carbohydrate metabolism and assimilation were identified (E value, 1 × 10^−4^), including glycoside hydrolases (*n* = 113), carbohydrate esterases (*n* = 77), glycosyl transferases (*n* = 76), enzymes involved in auxiliary activities (*n* = 46), carbohydrate-binding modules (*n* = 21), and polysaccharide lyases (*n* = 1). The Transporter Classification Database (TCDB; http://www.tcdb.org/) identified 283 major facilitator superfamily (MSF) transporters with an E value of 1 × 10^−5^, reflecting the ability of the BYSS01 isolate to extrude toxic compounds; efflux pumps have been shown to aid in adaptation to hydrocarbons in bacteria (11, 12).

The Kyoto Encyclopedia of Genes and Genomes (KEGG) database and BLASTP searches identified important proteins involved in carbon and energy metabolism (*n* = 49). In agreement with the ability of BYSS01 to grow in fuel, genes involved in the degradation of aromatics and *n*-alkanes, including cytochrome P450 alkane hydroxylase, cytochrome P450 monooxygenase, aromatic ring-opening dioxygenase, salicylate hydroxylase, 2-haloacid dehalogenase, benzyl alcohol dehydrogenase, benzoate 4-monooxygenase, and dimethyl-sulfide monooxygenase, were found. This genome sequence will help to understand the adaptive mechanisms employed by fungi to survive and proliferate in hydrocarbon-based fuels.

### Accession number(s).

This whole-genome shotgun project has been deposited at DDBJ/ENA/GenBank under the accession number NIXA00000000.

## References

[1] Kotzekidou P 1997 Heat resistance of *Byssochlamys nivea*, *Byssochlamys fulva* and *Neosartorya fischeri* isolated from canned tomato paste. J Food Science 62:410–413. doi:10.1111/j.1365-2621.1997.tb04014.x.

[2] Iwahara M, Fukuda R, Nakahara K, Tanaka T, Nomura Y 2001 Isolation and properties of Paecilomyces sp. no. 5 capable of degrading high concentrations of formaldehyde. BioControl Sci 7:107–110.

[3] Fukuda R, Nagahama K, Fukuda K, Ekino K, Oka T, Nomura Y 2012 Purification and properties of *S*-hydroxymethylglutathione dehydrogenase of *Paecilomyces variotii* no. 5, a formaldehyde-degrading fungus. AMB Express 2:32. doi:10.1186/2191-0855-2-32. 22731626 PMC3439253

[4] Oka T, Ekino K, Fukuda K, Nomura Y 2014 Draft genome sequence of the formaldehyde-resistant fungus *Byssochlamys spectabilis* no. 5 (anamorph *Paecilomyces variotii* no. 5) (NBRC109023). Genome Announc 2:e01162-13. doi:10.1128/genomeA.01162-13. 24407650 PMC3886963

[5] Bolger AM, Lohse M, Usadel B 2014 Trimmomatic: a flexible trimmer for Illumina sequence data. Bioinformatics 30:2114–2120. doi:10.1093/bioinformatics/btu170. 24695404 PMC4103590

[6] Bankevich A, Nurk S, Antipov D, Gurevich AA, Dvorkin M, Kulikov AS, Lesin VM, Nikolenko SI, Pham S, Prjibelski AD, Pyshkin AV, Sirotkin AV, Vyahhi N, Tesler G, Alekseyev MA, Pevzner PA 2012 SPAdes: a new genome assembly algorithm and its applications to single-cell sequencing. J Comput Biol 19:455–477. doi:10.1089/cmb.2012.0021. 22506599 PMC3342519

[7] Simão FA, Waterhouse RM, Ioannidis P, Kriventseva EV, Zdobnov EM 2015 BUSCO: assessing genome assembly and annotation completeness with single-copy orthologs. Bioinformatics 31:3210–3212. doi:10.1093/bioinformatics/btv351. 26059717

[8] Smit AFA, Hubley R, Green P RepeatMasker Open-3.0. 1996–2010 http://www.repeatmasker.org.

[9] Stanke M, Steinkamp R, Waack S, Morgenstern B 2004 AUGUSTUS: a web server for gene finding in eukaryotes. Nucleic Acids Res 32:W309–W312. doi:10.1093/nar/gkh379. 15215400 PMC441517

[10] Lombard V, Ramulu HG, Drula E, Coutinho PM, Henrissat B 2014 The carbohydrate-active enzymes database (CAZy) in 2013. Nucleic Acids Res 42:D490–D495. doi:10.1093/nar/gkt1178. 24270786 PMC3965031

[11] Gunasekera TS, Bowen LL, Zhou CE, Howard-Byerly SC, Foley WS, Striebich RC, Dugan LC, Ruiz ON 2017 Transcriptomic analyses elucidate adaptive differences of closely related strains of *Pseudomonas aeruginosa* in fuel. Appl Environ Microbiol 83:e03249-16. doi:10.1128/AEM.03249-16. 28314727 PMC5411511

[12] Ruiz ON, Brown LM, Striebich RC, Smart CE, Bowen LL, Lee JS, Little BJ, Mueller SS, Gunasekera TS 2016 Effect of conventional and alternative fuels on a marine bacterial community and the significance to bioremediation. Energy Fuels 30:434–444. doi:10.1021/acs.energyfuels.5b02439.

